# IgE-mediated cow’s milk allergy in patients under 2 years of age, suffering from AD

**DOI:** 10.1186/2045-7022-1-S1-P74

**Published:** 2011-08-12

**Authors:** Nino Lomidze, Maia Gotua, Tamar Gotua

**Affiliations:** 1 Center of Allergy & Immunology, Tbilisi, Georgia; 2 Jvania's Children Hospital, Tbilisi, Georgia

## Background

Food allergy affects 6-8% of children before the age of 5 and is a frequent cause of many allergic diseases. Sensitization in infancy predominantly occurs first to cow's milk and egg white. It is estimated that 2 to 5 % of infants develop milk allergy. Food allergy is a known provoking cause of Atopic Dermatitis (AD) in a subset of affected children and triggers skin symptoms in about 30% of children. The aim of our study was to evaluate the role of sensitization to cow's milk under 2 years of age with AD in Georgian population.

## Method

121 patients under 2 years of age with AD (male-60, female-61) were investigated. Analyzed data included clinical history, Severity Scoring of Atopic Dermatitis Index (SCORAD), measurements of total serum IgE and specific IgE to food allergens was tested by CAP System_ fluorescent enzyme immunoassay (ImmunoCap, Phadia). Specific IgE level ≥ 0.35 kUA/l was considered as positive.

## Results

Specific IgE to cow's milk was positive in 33.9% of cases. Among them the high level of allergen specific IgE to cow's milk (RAST classes 4-5) revealed in 2.4% of patients with atopic dermatitis, moderate level (3 class) - 4.9%, low level (1-2 classes) - 27.3%. Increased value of total IgE was detected in 45.4% of studied children. In some patients suspected having allergy to other food allergens, specific IgE to casein (29 investigated cases), to flour mixture - wheat, oat, maize, sesame, seed, buckwheat (32 cases), and to hen's egg (30 cases) were measured. Co-sensitization with milk was revealed in 20.7% of casein studied cases, 15.6% of flour mixture and 26.6% of hen's egg positive cases. 0.8% of infants with atopic dermatitis showed positive results to cow's milk, hen's egg and flour mixture. The degree of sensitization was correlating with SCORAD.

## Conclusion

Our study demonstrates that 33,9% of children under 2 years of age with AD in Georgia is sensitized to milk allergen, that should be considered for early induction of proper dietary, treatment and preventive measures. Further investigations are strongly recommended.

